# Diretrizes da Sociedade Brasileira de Cardiologia: Novas Normas, Novos Desafios

**DOI:** 10.36660/abc.20240258

**Published:** 2024-06-03

**Authors:** Leonardo Castro Luna, Helena Cramer Veiga Rey, Humberto Graner Moreira, José Airton de Arruda, Pedro Gabriel Melo de Barros e Silva, Mario de Seixas Rocha

**Affiliations:** 1 Hospital de Clínicas de Porto Alegre Porto Alegre Brasil Hospital de Clínicas de Porto Alegre, Porto Alegre – Brasil; 2 Universidade Federal do Rio Grande do Sul Porto Alegre Brasil Universidade Federal do Rio Grande do Sul, Porto Alegre – Brasil; 3 Hospital Moinhos de Vento Porto Alegre Brasil Hospital Moinhos de Vento, Porto Alegre – Brasil; 4 Instituto Nacional de Cardiologia Ministério da Saúde Rio de Janeiro RJ Brasil Instituto Nacional de Cardiologia, Ministério da Saúde, Rio de Janeiro, RJ – Brasil; 5 Faculdade de Medicina da Universidade Federal de Goiás Goiânia GO Brasil Faculdade de Medicina da Universidade Federal de Goiás, Goiânia, GO – Brasil; 6 Hospital Israelita Albert Einstein Unidade Goiânia Goiânia GO Brasil Hospital Israelita Albert Einstein - Unidade Goiânia, Goiânia, GO – Brasil; 7 Hospital Evangélico Vila Velha ES Brasil Hospital Evangélico, Vila Velha, ES – Brasil; 8 Hcor Research Institute São Paulo SP Brasil Hcor Research Institute, São Paulo, SP – Brasil; 9 Brazilian Clinical Research Institute São Paulo SP Brasil Brazilian Clinical Research Institute, São Paulo, SP – Brasil; 10 Centro Universitário São Camilo São Paulo SP Brasil Centro Universitário São Camilo, São Paulo, SP – Brasil; 11 Escola Bahiana de Medicina e Saúde Pública Salvador BA Brasil Pós-graduação em Medicina e Saúde Humana da Escola Bahiana de Medicina e Saúde Pública, Salvador, BA – Brasil; 12 Hospital Mater Dei Salvador BA Brasil Hospital Mater Dei, Salvador, BA – Brasil

**Keywords:** Guia, Guia de Prática Clínica, Publicações Científicas e Técnicas, Comunicação e Divulgação Científica

As diretrizes médicas desempenham um papel fundamental na orientação dos profissionais de saúde e na promoção de padrões de cuidados eficazes e seguros. Essas foram descritas há décadas e surgiram com o movimento da medicina baseada em evidências, sistematizando a melhor ciência disponível em recomendações práticas. A cardiologia foi pioneira nesse sentido, com a American Heart Association e o American College of Cardiology criando juntos, em 1980, uma força-tarefa para elaborar e fornecer orientações detalhadas e baseadas em evidências sobre o diagnóstico e tratamento de diversas condições cardiovasculares.^1^ Outro documento marcante pelo pioneirismo foi a diretriz para o tratamento da hipertensão arterial, publicada oficialmente pelo Joint National Committee dos Estados Unidos em 1977, que estabeleceu novos padrões de cuidado em uma época de rápidas mudanças científicas.^2^

Diretrizes médicas têm como objetivo maior o de refletir um esforço colaborativo entre especialistas, pesquisadores e organizações de saúde para sintetizar as mais recentes e melhores evidências científicas e traduzi-las em recomendações para a prática clínica.^3^ Ao longo dos anos, a elaboração das diretrizes médicas foi incorporando metodologias mais rigorosas e buscando maior transparência. O processo envolve uma revisão abrangente da literatura, identificação e avaliação crítica das evidências disponíveis, elaboração de recomendações precisas com base nesses achados e, por fim, revisão por pares e validação por especialistas.^4,5^ Além disso, as diretrizes geralmente são atualizadas periodicamente para refletir os avanços científicos e as mudanças que devem ter impacto nas práticas médicas.^6^

Vários estudos descreveram o impacto das diretrizes em indicadores de saúde e no atendimento direto aos pacientes. São vastas as evidências demonstrando que elas ajudaram a padronizar os cuidados de saúde, garantindo que os pacientes recebam os melhores tratamentos disponíveis. Em muitos cenários, a melhoria na qualidade dos cuidados foi associada à redução de complicações e mortalidade, além de contribuir para o uso mais eficiente dos recursos de saúde.^7-9^

Se por um lado as diretrizes trouxeram muitos avanços, por outro lado, a rápida evolução do conhecimento médico coloca um ônus sobre as sociedades científicas para atualizar continuamente as diretrizes, sendo um processo que exige tempo, diligência e recursos consideráveis. Paralelamente, o acesso às recomendações e a forma como são oferecidas constituem desafios únicos. A variação na infraestrutura de saúde, limitações de recursos, diferenças culturais e heterogeneidade na formação médica também são barreiras que reforçam a necessidade de uma abordagem flexível e contemporânea no oferecimento e disseminação das diretrizes.

## Diretrizes na Sociedade Brasileira de Cardiologia

As primeiras diretrizes da Sociedade Brasileira de Cardiologia (SBC) surgiram também nas décadas de 1980 e 1990, abordando as questões relacionadas ao diagnóstico e tratamento das doenças cardiovasculares prevalentes, como hipertensão arterial, doença arterial coronariana e insuficiência cardíaca.^10^ Em 2006, foi instituído o Conselho de Normatizações e Diretrizes (ConDir) da SBC, responsável por coordenar a elaboração de diretrizes e normas técnicas para orientar a prática clínica dos cardiologistas brasileiros. Assim como para outras sociedades internacionais de cardiologia, esses documentos têm metas claras, e incluem: melhorar a qualidade da assistência prestada; contribuir para que tratamentos seguros e eficazes possam ser oferecidos ao maior número possível de pacientes, independentemente do local e do profissional de saúde que o atendeu; evitar práticas inapropriadas e não respaldadas cientificamente; e, finalmente, otimizar o uso dos recursos disponíveis, evitando desperdícios e buscando manter a sustentabilidade do sistema de saúde.

Certamente, as Diretrizes da SBC se tornaram uma referência importante para o atendimento dos pacientes com doenças cardiovasculares no Brasil, sendo não só um balizador aos profissionais de saúde, mas também um norte a todo ecossistema: prestadores, fontes pagadoras e gestores em saúde.

## Nova proposta para SBC

Ao longo dos anos, os membros do ConDir, dessa e de gestões passadas, se depararam com os desafios para aprimorar os documentos científicos da SBC. Ouvindo a comunidade, quem coordena, escreve, divulga, e especialmente os médicos que leem e usam as diretrizes na prática, foi iniciado um processo de reformulação das normativas. Nesse processo, o ConDir, em alinhamento com as recomendações do Guidelines International Network (GIN),^11^ GIN-McMaster Guideline Development Checklist^12-14^ e outros organismos internacionais, estabeleceu uma atualização das suas normas e sua composição, as quais foram homologadas pelo Conselho Diretivo da SBC e recentemente publicadas.^15^ As novas normas trazem mudanças estruturais significativas no modelo de desenvolvimento destes documentos.

As normas têm 3 diferenças importantes em relação às regras anteriores: 1) redefinição dos termos diretrizes e posicionamento; 2) a introdução de recomendações médicas, documentos menores e objetivos, elaborados a partir de uma revisão sistemática e com a finalidade de responder uma pergunta científica no formato PICO (P = população; I = intervenção; C = comparador; O = desfecho); 3) a adoção do Sistema GRADE (Grading of Recommendations Assessment, Development and Evaluation) para estabelecer o nível de evidência e força da recomendação (Quadro 1).

**Quadro 1:** O que muda nas novas normativas das Diretrizes da SBC

Até 2023	A partir de 2024
Terminologia: diretrizes, posicionamentos e atualizações	Terminologia: recomendações, diretrizes e posicionamentos
Coordenadores e especialistas escolhidos por tema ou subitem da Diretriz	Coordenadores e líderes definem escopo da Diretriz e elaboram perguntas PICO
Revisão da literatura pelos especialistas	Revisão sistemática da literatura por metodologistas e especialistas
Força de recomendação e nível de evidência baseados nos artigos disponíveis da literatura sobre o tema	Força de recomendação e nível de evidência baseados no conjunto das evidências por desfecho clínico
**Graduação da recomendação em categorias de 1, 2a, 2b e 3, com força da evidência classificada em A, B ou C.**	**Graduação mais simples (recomendação forte, fraca ou neutra; certeza de evidência alta, moderada, baixa ou muito baixa) Sistema GRADE para estabelecer o nível de evidência e força da recomendação**
Documentos mais extensos abordando epidemiologia, diagnóstico e tratamento de forma textual	Documentos mais curtos com perguntas PICO relevantes e recomendações objetivas da SBC sobre o tema
	Exposição do tamanho do efeito (desfechos de benefício e malefício) através de tabelas
Realização de nova diretriz com intervalos de alguns anos	Realização periódica da busca de cada Recomendação para avaliar a necessidade de atualização
Recomendação médica definida por especialistas no tema	Recomendação médica definida por um painel de profissionais diverso e multidisciplinar
Avaliação dos conflitos de interesse financeiros de cada participante	Avaliação dos conflitos de interesse financeiros e intelectuais de cada participante
	Priorização de desfechos clínicos críticos e importantes para o paciente, evitando-se desfechos não importantes ou substitutos

**Figura 1:**
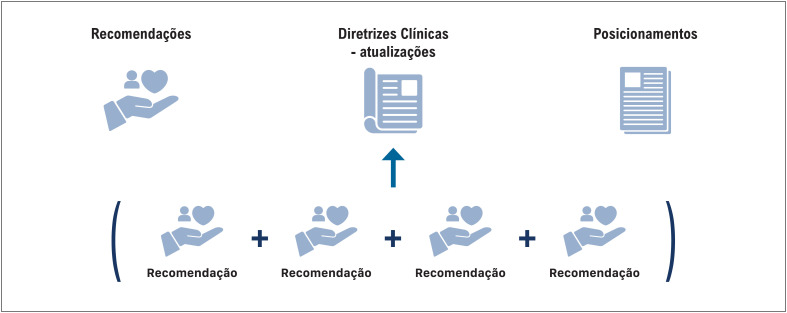
Documentos científicos da Sociedade Brasileira de Cardiologia.

Os posicionamentos (*statement*) e suas diferenças em relação a uma diretriz (*guidelines*) são destacados nessas orientações. As diretrizes clínicas são destinadas a orientar tecnicamente os profissionais de saúde e população em relação a determinada condição de saúde ou tecnologia. Os posicionamentos são focados em documentos de natureza normativa, regulatória ou de ensino da SBC. Nessa construção foi inserido um novo informativo denominado de "recomendação", que é o elemento base para a construção das diretrizes (Figura 1). Este novo documento se destina a dar suporte aos profissionais em uma tomada de decisão específica e se baseia numa revisão sistemática da literatura de acordo com a pergunta PICO escolhida, e que pode ser elaborada pela própria SBC ou utilizando uma revisão sistemática já publicada, incluindo uma adaptação de uma diretriz clínica atual de outra instituição sobre o tema. Após a apresentação dos dados científicos desta revisão sistemática, um painel diverso e multidisciplinar gerará uma recomendação sob o formato GRADE (força de recomendação e certeza da evidência científica). As recomendações serão publicadas no formato de artigo original pelo periódico Arquivos Brasileiros de Cardiologia (ABC Cardiol), e cada recomendação subsidiará, no futuro, as atualizações das diretrizes plenas. Dessa forma, busca-se que as novas diretrizes sejam baseadas nas melhores evidências científicas disponíveis utilizando métodos reconhecidos, robustos e transparentes. Além disso, as novas normas reiteram que uma das premissas fundamentais das diretrizes clínicas é que elas sejam desenvolvidas levando em consideração as opiniões daqueles que podem ser afetados pela diretriz (incluindo profissionais de saúde e outros profissionais, pacientes e seus cuidadores, gestores do sistema de saúde, órgãos governamentais e o sistema suplementar de saúde).

Finalmente, as novas normas da SBC estabelecem que a apresentação de uma recomendação clínica deve seguir a metodologia GRADE.^13,14^ No novo modelo de apresentação, estarão bem estabelecidas a direção (contra ou a favor) e a força da recomendação (forte ou fraca), além do nível de certeza das evidências indicado pelo metodologista (alto, moderado, baixo ou muito baixo). O modelo de redação a ser adotado para as recomendações é: "Para a população X, a Sociedade Brasileira de Cardiologia recomenda (a favor ou contra) a adoção da estratégia Y (em detrimento da estratégia Z - opcional); recomendação (forte ou fraca) baseada em um nível de certeza (alto, moderado, baixo ou muito baixo) da evidência científica.".

**Figura 2:**
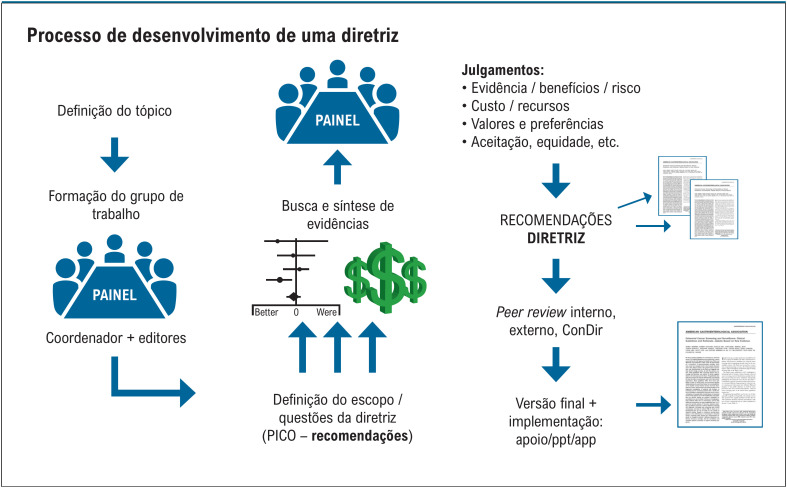
Ciclo para desenvolvimento das Diretrizes da SBC. ConDir: Conselho de Normatizações e Diretrizes.

No sentido de garantir a melhor relação entre todas as partes envolvidas, as normas descrevem também os papéis e responsabilidades de todos os envolvidos nas construções destes documentos, incluindo os membros do Conselho de Normatização de Diretrizes (CONDIR), o coordenador(a) de diretriz, o editor(a) de recomendações, os membros do painel de recomendação, o metodologista, o grupo revisor e os membros de integração (Figura 2). Desta maneira, busca-se trazer clareza para a organização das atividades e, principalmente, transparência, reprodutibilidade e maior segurança de que as decisões do painel se apoiam nas melhores evidências.

As relações entre o negócio da saúde, indústria farmacêutica, equipamentos, órteses e próteses sempre permearão a existência de uma sociedade médica, mas é consenso dentro da condução de qualquer ato médico que exista transparência e ética. As normas da Diretrizes da SBC sempre foram explícitas em relação à importância da declaração de conflito de interesse entre seus participantes, e a revisão das normativas reforça e corrobora as definições vigentes.

## Reflexões futuras, desafios e novas perspectivas

O período de transição rumo a um modelo renovado de diretrizes ressalta a importância da flexibilidade e do apoio nessa fase em que documentos antigos e novos irão conviver. A SBC adota, então, uma abordagem híbrida, permitindo a integração de elementos do modelo anterior com inovações, como as perguntas PICO e a aplicação do sistema GRADE. Tal modelo híbrido permitirá uma transição gradual, embora traga consigo desafios específicos de integração e clareza. Neste momento, a SBC está estruturada para dar apoio aos líderes dos documentos na condução e elaboração destes, bem como buscar novos formatos e mecanismos de divulgação e comunicação com a comunidade médica.

A implementação efetiva das diretrizes apresenta obstáculos notáveis, tais como a resistência à mudança, a heterogeneidade nas práticas médicas a nível regional e os entraves no acesso a tecnologias inovadoras e tratamentos avançados. A frequência das atualizações, por sua vez, pode suscitar dúvidas entre os profissionais da saúde. Diante desses desafios, a SBC tem adotado medidas estratégicas, incluindo iniciativas de educação médica continuada e o desenvolvimento de ferramentas digitais para promover o acesso facilitado às diretrizes revistas.^15^

O processo de transição nas diretrizes da SBC é um testemunho do comprometimento da Sociedade com a prática baseada em evidências e com o aprimoramento contínuo do cuidado ao paciente. Embora represente um desafio, este período é crucial para o progresso da cardiologia no Brasil. Com o suporte ininterrupto da SBC, a adaptabilidade durante a transição e a adesão às metodologias atualizadas, a cardiologia brasileira está destinada a avançar com excelência e pertinência.
