# Supplementary material for: Diretrizes da Sociedade Brasileira de Cardiologia: Novas Normas, Novos Desafios
Source: Arq Bras Cardiol. 2024 Jun 3;121(6):e20240258. [Article in Portuguese] doi: 10.36660/abc.20240258 (PMC11156215; doi:10.36660/abc.20240258)
Supplement: Translation

# Translation: Brazilian Society of Cardiology Guidelines: New Norms, New Challenges

**DOI:** 10.36660/abc.20240258i

**Authors:** Leonardo Castro Luna, Helena Cramer Veiga Rey, Humberto Graner Moreira, José Airton de Arruda, Pedro Gabriel Melo de Barros e Silva, Mario de Seixas Rocha

**Affiliations:** 13 Porto Alegre Brazil Hospital de Clínicas de Porto Alegre, Porto Alegre – Brazil; 2 Porto Alegre Brazil Universidade Federal do Rio Grande do Sul, Porto Alegre – Brazil; 3 Porto Alegre Brazil Hospital Moinhos de Vento, Porto Alegre – Brazil; 4 Rio de Janeiro RJ Brazil Instituto Nacional de Cardiologia, Ministério da Saúde, Rio de Janeiro, RJ – Brazil; 5 Goiânia GO Brazil Faculdade de Medicina da Universidade Federal de Goiás, Goiânia, GO – Brazil; 6 Goiânia GO Brazil Hospital Israelita Albert Einstein - Unidade Goiânia, Goiânia, GO – Brazil; 7 Vila Velha ES Brazil Hospital Evangélico, Vila Velha, ES – Brazil; 8 São Paulo SP Brazil Hcor Research Institute, São Paulo, SP – Brazil; 9 São Paulo SP Brazil Brazilian Clinical Research Institute, São Paulo, SP – Brazil; 10 São Paulo SP Brazil Centro Universitário São Camilo, São Paulo, SP – Brazil; 11 Salvador BA Brazil Pós-graduação em Medicina e Saúde Humana da Escola Bahiana de Medicina e Saúde Pública, Salvador, BA – Brazil; 12 Salvador BA Brazil Hospital Mater Dei, Salvador, BA – Brazil

**Keywords:** Guideline, Practice Guideline, Scientific and Technical Publications, Scientific Communication and Diffusion

Medical guidelines play a critical role in guiding health care professionals and promoting effective and safe standards of care. They emerged decades ago starting with the evidence-based medicine movement, systematizing the best available science into practical recommendations. Cardiology was a pioneer in this regard: in 1980 the American Heart Association and the American College of Cardiology formed a joint task force to develop and provide detailed, evidence-based guidance on diagnosis and treatment for various cardiovascular conditions.^1^ Another pioneering document was a high blood pressure treatment guideline by the U.S. Joint National Committee in 1977, which set new care standards in a time of rapid scientific change.^2^

Medical guidelines are a collaborative effort between experts, researchers, and health care organizations to synthesize the latest and best scientific evidence and translate it into recommendations for clinical practice.^3^ Over the years, medical guidelines have incorporated more rigorous methodologies and sought greater transparency. The process involves a comprehensive review of the literature, identification and critical assessment of the available evidence, the development of precise recommendations based on these findings, and finally, peer review and validation by experts.^4,5^ Generally, guidelines receive periodic updates to reflect scientific advances and changes that are expected to impact medical practice.^6^

A number of studies have described the impact of guidelines on health indicators and direct patient care. There is extensive evidence that they have helped standardize health care, ensuring that patients receive the best available treatment. Improved care quality has been associated with a reduction in complications and mortality, in addition to more efficient use of health resources.^7-9^

While, on the one hand, guidelines have brought many advances, on the other hand, the quick evolution of medical knowledge requires scientific societies to continually update them, a process that demands considerable time, diligence, and resources. Moreover, the way in which recommendations are presented and ensuring access to them entail unique challenges. Variation in health care infrastructure, resource limitations, cultural differences, and heterogeneous medical training also call for a flexible and contemporary approach to the development and dissemination of guidelines.

## Brazilian Society of Cardiology Guidelines

The first Brazilian Society of Cardiology (SBC) guidelines also emerged in the 1980s and 1990s, addressing issues related to the diagnosis and treatment of prevalent cardiovascular diseases, such as high blood pressure, coronary artery disease, and heart failure.^10^ In 2006, the SBC's Clinical Practice Guidelines Committee (ConDir) was established. It is responsible for coordinating the development of guidelines and technical standards for clinical practice among Brazilian cardiologists. The guidelines of other international cardiology societies have clear goals, including: improved care quality; safe and effective treatment for the greatest number of patients, regardless of location or health care provider; curbing unscientific practices; and optimal use of the available resources, avoiding waste and promoting the health system's sustainability.

SBC Guidelines have become an important reference for cardiovascular care in Brazil, serving as a guide for health professionals and the entire ecosystem: providers, payment sources, and health managers.

## New proposal for the Brazilian Society of Cardiology

ConDir members from current and past administrations have faced challenges in improving the SBC's scientific documents. Through listening to the community, ie, coordinators, writers, disseminators, and especially doctors who read and use the guidelines, a revision process was initiated. ConDir, in alignment with the Guidelines International Network (GIN),^11^ the GIN-McMaster Guideline Development Checklist,^12-14^ and other international organizations, updated its norms, which were approved by the SBC board of directors and recently published.^15^ The new standards have resulted in significant structural changes to the documents’ development model.

There are 3 important differences in relation to previous norms: 1) redefinition of the terms "Guidelines" and "Statements": 2) the introduction of Medical Recommendations, which are smaller, objective documents based on systematic reviews to answer a specific scientific question through the PICO (P = Population, I = Intervention, C = Comparator, O = Outcome) format; 3) and use of the Grading of Recommendations Assessment, Development and Evaluation (GRADE) System to indicate the strength and level of evidence of recommendations (chart 1).

**Chart 1:** What has changed in the new Brazilian Society of Cardiology Guidelines?

Up to 2023	From 2024
Terminology: Guidelines, Statements and Updates	Terminology: Recommendations, Guidelines and Statements
Coordinators and experts chosen by topic or sub-item of the Guideline	Coordinators and leaders define the scope of the Guideline and prepare PICO questions
Literature review by experts	Systematic literature review by methodologists and experts
Strength of Recommendation and Level of Evidence based on available literature articles on the topic	Strength of Recommendation and Level of Evidence based on the set of evidence by clinical outcome
**Graduation of the recommendation into categories of 1, 2a, 2b and 3, with strength of evidence classified as A, B or C.**	**Simpler grading (Strong, Weak, or Neutral Recommendation; High, Moderate, Low or Very Low Certainty of Evidence) GRADE system to establish the level of evidence and strength of the recommendation**
More extensive documents covering epidemiology, diagnosis and treatment in textual form	Shorter documents with relevant PICO questions and objective SBC recommendations on the topic
	Exposure of the effect size (benefit and harm outcomes) through Tables
Creation of new Guidelines every few years	Periodically carry out a search for each Recommendation to assess the need for updating
Medical recommendation defined by experts on the subject	Medical recommendation defined by a diverse and multidisciplinary panel of professionals
Assessment of each participant's Financial Conflicts of Interest	Assessment of each participant's Financial and Intellectual Conflicts of Interest
	Prioritization of critical and important Clinical Outcomes for the patient, avoiding unimportant or substitute outcomes

Regarding the difference between Statements and Guidelines, Clinical Guidelines are intended as a technical guide about a specific health condition or technology for health professionals and the public, whereas Statements refer to SBC documents of a normative, regulatory, or educational nature. Within this framework, Recommendations were added, which are the basic components of the Guidelines (Figure 1). This new document, which is intended to help professionals with specific decisions, is based on a systematic review of the literature according to the selected PICO question, and can be produced by the SBC itself or based on a previously published systematic review, including adaptations of current clinical guidelines from another institution. After presenting the systematic review data, a multidisciplinary panel will formulate a recommendation using the GRADE format (strength of recommendation and certainty of scientific evidence). Recommendations will be published as original articles in the *Arquivos Brasileiros de Cardiologia* and each Recommendation will be used in future updates of the Guidelines. Thus, new guidelines will be based on the best available evidence using recognized, robust, and transparent methods. According to new norms, clinical guidelines must be developed considering the views of those who may be affected by them, including health care and other professionals, patients and their caregivers, health care system managers, government agencies, and the supplementary health system.

**Figure 1:** Brazilian Society of Cardiology scientific documents.

Finally, according to new SBC norms, Clinical Recommendations must follow GRADE methodology.^13-14^ In the new model, the direction (against or for) and strength (strong or weak) of the recommendation will be presented, in addition to the level of evidence (high, moderate, low, or very low). The model for Recommendations is: "For population X, the SBC recommends (against or for) strategy Y based on a (high, moderate, low, or very low) level of scientific evidence".

To ensure the best relationship between all involved parties, the norms also describe the roles and responsibilities of everyone involved in the development of these documents, including the members of ConDir, the Guideline Coordinator, the Recommendation Editor, the Recommendation Panel Members, the Methodologist, the Review Group, and other team members (Figure 2). Thus, the aim is to maximize the organization of the process, especially its transparency and reproducibility, which will lead to greater certainty that decisions have been made according to the best evidence.

**Figure 2:** Development flowchart for the Brazilian Society of Cardiology Guidelines.

The relationship between the health care system, the pharmaceutical industry, equipment, orthotics, and prosthetics is a perpetual issue in medical societies, but there is consensus that transparency and ethics are essential for any medical act. The SBC Guidelines have always stressed the importance of declaring conflicts of interest between its participants, and the revised norms reinforce and corroborate current definitions.

## Future reflections, challenges and new perspectives

Flexibility and support are important during the transition phase to a new guideline model, in which both old and new documents will coexist. The SBC has adopted a hybrid approach, allowing the integration of elements from the previous model with innovations, such as PICO questions and the GRADE system. This hybrid model will allow for a gradual transition, although it involves certain challenges regarding integration and clarity. At this point, the SBC is supporting leaders in document development and is seeking new formats and mechanisms of dissemination and communication with the medical community.

There are serious obstacles to effective implementation of the guidelines, such as resistance to change, the heterogeneity of medical practice on a regional level, and limited access to innovative technologies and advanced treatments. The frequency of updates could also reduce confidence among health professionals. Due to these challenges, the SBC has adopted strategic measures, including continuing education initiatives and the development of digital tools, to promote easier access to the revised guidelines.

The transition process in SBC guidelines is a testament to the Society's commitment to evidence-based practice and continuous improvement in patient care. Although it is challenging, this period is critical for the progress of cardiology in Brazil. With SBC's uninterrupted support, adaptability during the transition period, and adherence to updated methodologies, Brazilian cardiology is destined to advance with excellence and relevance.
